# Comparison between Smoking and Nonsmoking Palestinian Medical Students in the Health-Promoting Behaviors and Lifestyle Characteristics

**DOI:** 10.1155/2021/5536893

**Published:** 2021-03-16

**Authors:** Nihad Al-Othman, Mustafa Ghanim, Moath Alqaraleh

**Affiliations:** ^1^Faculty of Medicine and Health Sciences, An-Najah National University, Nablus, State of Palestine; ^2^Pharmacological and Diagnostic Research Center (PDRC), Faculty of Pharmacy, Al-Ahliyya Amman University, Amman 19328, Jordan

## Abstract

**Objectives:**

This study was aimed at comparing the predictors of health-promoting lifestyle behaviors between smoking and nonsmoking medical students at An-Najah National University located in Palestine.

**Methods:**

A descriptive cross-sectional study was performed during the academic year 2017/2018. Medical students were asked to complete a self-reported questionnaire that involved the predictors of Health-Promoting Lifestyle Profile-II. With the use of a suitable available sample composed of a total of 430 medical students, 400 had successfully completed the questionnaire and were included in the study. The data was analyzed by using SPSS version 24 software.

**Results:**

The sample included 400 medical students with a mean age of 18.7 years, 311 (77.7%) were females, and 89 (22.3%) were males. The prevalence of smokers in the sample was 110 (27.5%). For the health status of over half the students, 211 (52.8%) were excellent. The total HPLP-II score for smoking students resulted to be significantly lower in comparison to nonsmoking students (131.2 versus 135.7). This significant difference was clear in the interpersonal relation subscale (25.6 versus 26.8) for smoking and nonsmoking students, respectively. The score differences in other subscales were generally lower in smoking students. However, these differences were not consistent with statistical significance.

**Conclusion:**

The significant lower total Health-Promoting Lifestyle Profile-II score in smoking students necessitates the urgent need for awareness programs, not only towards smoking but also on how to enhance student health-promoting lifestyle behaviors.

## 1. Introduction

Smoking is considered a central public health problem as it is among the leading preventable causes of death worldwide [1]. It is estimated that more than 8 million people die every year as a direct or indirect result of smoking [2]. Around 80% of smokers reside in low- and middle-income countries, which further aggravates the deleterious health effects associated with tobacco in these countries, especially that they lack sufficient resources to adopt preventive and management programs against smoking [2, 3]. Palestine has very limited income resources and suffers from the continuity of the rising rate of smokers, particularly among university students [4–6]. Such status can be quite frustrating because it is reported that the earlier the smoking intake, the more susceptible an individual can be for cancer, heart disease, and other health-related consequences [7, 8]. Previous studies in Arab countries, including Palestine, focused on the estimation of the prevalence, awareness, patterns, and attitudes of smoking [3, 9, 10]. A recent study found that Palestinian smoking university students adapted towards unhealthy habits such as consuming high amounts of caffeinated drinks as well as fast food, which would accelerate the occurrence and seriousness of smoking health effects [4]. Some studies within the Arabian Peninsula and nearby countries addressed the unhealthy behaviors and lifestyle among university students using the Pender model [9, 10]. This model focused on a lifestyle that triggers the consumption of a low-fat diet, performing consistent physical activities, preserving healthy body weight, and preventing smoking and anxiety in youth [11].

The objective of this study was to assess the differences in health-promoting behaviors and lifestyle between smoking and nonsmoking medical students at An-Najah National University in Palestine with the use of the Pender model.

## 2. Materials and Methods

### 2.1. Study Sample

A cross-sectional study was done to investigate the health-promoting lifestyle behaviors (HPLBs) among students at the Faculty of Medicine and Health Sciences at An-Najah National University, the largest university in Palestine, during the spring semester of 2018. A convenient nonprobability available sample took part in this study. The size of the sample was estimated using Jekel et al. equation [12]. Assuming the probability of smoking among students is 0.5 with a confidence level of 95%, the estimated minimal sample size was 384. Nevertheless, we decided to increase the sample size to 431 in order to decrease the standard error of the mean and to account for nonresponse rate. Only 400 students who were included in the study agreed voluntarily to complete the questionnaire for research purposes. We excluded 31 students who failed to complete the questionnaire or did not sign the consent form.

### 2.2. Study Instrument

We used the previously validated self-administered questionnaire: Health-Promoting Lifestyle Profile-II (HPLP-II), after we had got the official permission from the author who invented this tool. HPLP-II is an extensively useful tool that helps researchers to measure the patterns and determinants of HPLBs [13]. The questionnaire was distributed to the students in English which is the official adopted language in studying medicine in the faculty. The HPLP-II is composed of a 52-item questionnaire which is made of two main categories (health-promoting behaviors and psychosocial well-being) and six subscales as it is explained in Table 1. Participants answer each question of the 52 items on a four-point Likert scale (1, never; 2, sometimes; 3, and often 4, routinely). The subscale score was calculated by summing the scores for all items within that subscale. The total HPLP-II score was calculated as the sum of all subscale scores, with a higher score being consistent with better HPLBs.

### 2.3. Pilot Study

Researchers performed a pilot study composed of 20 undergraduate medical students to examine the clarity and relevance of the instrument. The Cronbach alpha value for total HPLP-II was 0.881.

### 2.4. Ethical Approval

Informed signed consent has been acquired from all participants. This study got official ethical approval by the Institutional Review Board of research ethics according to the laws of An-Najah National University located in Nablus, Palestine.

### 2.5. Statistical Analysis

Statistical analysis was done using SPSS version 24 software. A chi-square test was applied to compare the differences in item responses between smoking and nonsmoking medical students. An independent sample *t*-test was applied to analyze the variations in the total HPLP-II mean scores between the two groups. The *p*-value < 0.05 was considered statistically significant.

## 3. Results

### 3.1. Characteristics of the Sample

The age of the students ranged from 17 to 21 years (mean age was 18.7 years). Of the 400 students included in this study, 110 (27.5%) were smoking and 290 (72.5%) were nonsmoking. Data were tested by the normally test and were found to be normally distributed. As for the gender of students, 311 (77.7%) were females and 89 (22.3%) were males. About 223 (55.8%) of the students were in their first academic year. Over 69.3% of students were living in cities; 21.3% and 9.6% came from rural backgrounds and refugee camps, respectively. For the health status of over half of the students, 211 (52.8%) was excellent while it was good for 183 (45.8%) and bad for 6 (1.4%).

### 3.2. Health-Promoting Behaviors of Students

When analyzing health-promoting behaviors, only those who reported often or routinely engaging in each health practice item were considered practicing health-promoting behaviors. Those who reported never or sometimes were considered not practicing particular health-promoting behaviors.  Table 2 illustrates the results of the three subscales of health-promoting behaviors (health responsibility, physical activity, and nutritional habits) and the comparison between smoking and nonsmoking students who were considered practicing health-promoting behaviors.

#### 3.2.1. Health Responsibility (HR)

Only 47.9% of the students had a sense of overall HR. We found 19.2% of the students examining their body shape monthly for physical alterations or danger marks. About 38.3% of students watch TV programs about developing health, and 42.8% of them discuss health concerns with health professionals. The overall proportion of HR items among nonsmoking and smoking students was approximately equal. More nonsmoking than smoking students examine their body shape monthly for physical alterations or danger marks (24.8% versus 17.3%), attend learning programs on individual healthcare (37.6% versus 35.5%), follow TV programs about developing health (39% versus 36.4%), and discuss health fears with health professionals (45.2% versus 39.4%). On the other hand, fewer nonsmoking students seek advice from health professionals (59% versus 70.9%). Chi-square test analysis showed that, with the exception of the difference in the item (seeking advice from health professionals), all other differences regarding HR items between smoking and nonsmoking students were statistically insignificant.

#### 3.2.2. Physical Activity (PA)

On average, 25.1% of the students were involved in doing the PA items. Among the 400 students of the sample, 18% followed a designed exercise program, 26.5% were trained forcefully, 27.5% practiced light-to-moderate PA, and 19.3% inspected their target pulse rate while exercising. Nonsmoking and smoking students engaged equally in the overall PA items, whereas more nonsmoking than smoking students were trained forcefully for at least 20 minutes (27.9% versus 22.7%). Chi-square test shows that there were no statistically significant differences between the PA items and the smoking status.

#### 3.2.3. Nutritional Habits (NH)

About 40.3% of the students were involved in appropriate NH within the overall items. Only 29.8% of the students chose a diet poor in fat whether saturated or unsaturated, 34.3% restricted utilizing sugars and sweets, 36.5% consumed at least two to five servings of fruit, and 39% drank milk daily. More nonsmoking than smoking students had better NH (41.2% versus 38.7%), consumed vegetables (40.3% versus 34.5%), drank milk (40.7% versus 34.5%), and examined labels to discover nutrients, fats, and sodium in enclosed food (43.1% versus 38.1%). There were no statistically significant differences between NH items and the smoking status.

### 3.3. Psychosocial Well-Being

When analyzing the psychosocial well-being of the students, only those who reported often or routinely adopting a psychosocial practice were considered having psychological well-being.

Table 1 illustrates the results of the three subscales of psychosocial well-being (spiritual growth, interpersonal relations, and stress management) and the comparison between smoking and nonsmoking students who were considered having psychological well-being.

#### 3.3.1. Spiritual Growth (SG)

About 73.8% of the students resulted to have good SG within the overall items. More nonsmoking than smoking students had more efficient SG (74.9% versus 71.9%), felt that their existence has a purpose (84.5% versus 80%), and discovered that each day is exciting and challenging (60.7% versus 52.7%). Chi-square tests showed that there were no statistically significant differences between SG items and the smoking status.

#### 3.3.2. Interpersonal Relations (IR)

About 69.5% of the students had effective IR in the overall items (70.5% in nonsmoking versus 66.9% in smoking students). Statistically significant differences between nonsmoking and smoking students were found only in two items: discovering ways to meet their demands for intimacy (64.8% versus 53.6%) and talking more about their troubles and concerns to individuals close to them (62.8% versus 50%).

#### 3.3.3. Stress Management

Less than half of the students could manage stress in the overall items. A greater number of nonsmoking students showed stress-management skills (50.4% versus 48.2%) and obtained enough time for leisure each day (50.7% versus 43.6%), without statistical significance.

### 3.4. Comparison of Subscale Scores of the Health Promotion Lifestyle Profile II

Nonsmoking students had significantly higher total HPLP-II scores than smoking students (*t* = 1.702, *p* < 0.05). Nonsmoking students were shown to be significantly more actively engaged in IR subscale in comparison to smoking students (*t* = 2.011, *p* < 0.05). The subscale scores for HR, NH, SG, and SM were higher in nonsmoking students compared to smoking ones without statistical significance (Table 3).

## 4. Discussion

This is the first study to explore the differences in HPLBs among smoking and nonsmoking medical students in Palestine according to Pender's model. Medical students were the focus of this study as they represent the prospected doctors to serve people in the country. The results of this study help the stakeholders at the universities for better understanding of the health habits of medical students in order to ameliorate their future responsibility towards their humanitarian professions.

This study found that smoking students resulted to have a significantly lower total mean value for HPLP-II than that in nonsmoking students (*t* = 1.702, *p* < 0.05). Based on this, we can conclude that it appears that smoking students show less care about their health and nutrition, despite their realization of the deleterious health effects of smoking on their well-being [10]. These findings are consistent with previous regional and international studies [10, 14–16] but are inconsistent with other studies [17].

This study revealed that the total HPLP-II mean score for medical students was 133.4, which is higher than that observed in Saudi Arabia [14], Turkey [10], and Iran [18]. These variations in HPLP-II mean scores could result from including nursing, paramedical, or health sciences students in these studies while our sample included medical students. The high total mean scores of HPLP-II for medical students reflect their high level of health awareness, which is attributed to the nature of their health and scientific background as prospective doctors [19, 20].

Our results showed that the differences between smoking and nonsmoking medical students were insignificant in all HPLP-II subscales with the exception of the IR subscale which was significantly lower in nonsmoking students. This is consistent with other studies [10, 21, 22]. These findings are assuring that smoking will not significantly affect most of the HPLB items. With this regard, it seems that smoking decreases the chances of students to talk about their troubles and concerns and reduces their skills to discover ways to meet their demands for intimacy.

Our research revealed that 27.5% of medical students resulted to be smokers. These findings are in agreement with previous studies [22–24]. Regarding the gender, there were more smoking males compared to females. The gender-specific smoker frequency in our study was lower than what was reported in previous studies in Palestine [23], Syria [25], and Saudi Arabia [26]. The lower ratio of female smokers is related to traditions and cultural backgrounds, as smoking is still considered a social stigma for females [27, 28]. It is noteworthy that there was a female predominance in our sample which reflects the high ratio of female students at the faculty. This might have affected the results concerning the gender.

There were several limitations of this study. The convenient sample was obtained from a single university which is the largest university in Palestine. Yet, it may not be representative of all Palestinian medical students. Additional thorough studies including medical students from all other universities in Palestine are needed. The questionnaire was self-reported, and so reporting bias cannot be excluded. The questionnaire was administered to students during their regular classes which may have influenced the responses, and students who were absent on that day did not have the chance to participate. We did not specify if the form of smoking is cigarette, water pipe, or cigar.

## 5. Conclusions

This study provides new information about the possible impact of smoking status among medical students on the performance of HPLBs. A significant difference level of the total HPLP-II mean score was found between smoking and nonsmoking university students. No statistically significant differences were found between the two groups and all subscales except for IR.

University educators should acquire a perception of the significance of including the concepts of HPLBs and lifestyle modifications within the curriculum plan. It is vital to implement the programs of smoking cessation among medical students as they represent future doctors. Further studies with more representative samples from different faculties and various universities are needed for a better understanding of the impact of smoking on different HPLB scores.

## Figures and Tables

**Table 1:** Comparison of psychosocial well-being between smoking and nonsmoking medical students (*n* = 400) by the chi-square test.

Subscale	Smoking (*n* = 110) *N* (%)	Nonsmoking (*n* = 290) *N* (%)	Total *N* (%)	*X* ^2^	*p* value
Spiritual growth					
Q27: I believe I am growing and varying in positive ways	72 (65.45)	189 (65.17)	261 (63.3)	0.00	0.95
Q28: I feel that my existence has a purpose	88 (80)	245 (84.482)	333 (83.3)	1.15	0.28
Q29: I look ahead to the future	93 (84.54)	253 (87.24)	346 (86.5)	0.49	0.48
Q30: I feel comfortable and in harmony with myself	84 (77.06)	216 (74.48)	300 (75)	0.28	0.59
Q31: I work in the direction of long-term aims in my life.	86 (78.18)	234 (80.68)	320 (80)	0.31	0.57
Q32: I discover that every day is exciting and challenging	58 (52.72)	176 (60.68)	234 (58.5)	2.08	0.15
Q33: I am attentive to what is vital to me in my life	85 (77.27)	235 (81.03)	320 (80)	0.70	0.40
Q34: I believe linked with some force larger than myself	70 (63.63)	207 (71.38)	277 (69.3)	2.24	0.13
Q35: I expose me to new skills in addition to challenges	75 (68.18)	199 (68.62)	274 (68.5)	0.01	0.93
*Mean (%)*	71.9	74.9	73.8		
Interpersonal relations					
Q36: I talk about my troubles and concerns with individuals close to me	55 (50)	182 (62.75)	237 (59.3)	5.37	0.02^∗^
Q37: I compliment other individuals simply for their successes	76 (69.09)	202 (69.65)	278 (69.5)	0.01	0.91
Q38: I keep important and satisfying relationships with others	87 (79.09)	236 (81.38)	323 (80.7)	0.26	0.60
Q39: I spend some time with my close friends	82 (74.54)	217 (74.82)	299 (74.8)	0.00	0.953
Q40: I simply show worry, love, and warmth to many individuals	60 (54.54)	185 (63.79)	245 (61.3)	2.87	0.09
Q41: I touch and I am touched by individuals that I concern about	83 (75.45)	215 (74.14)	298 (74.5)	0.07	0.79
Q42: I discover ways to meet my demands for intimacy	59 (53.63)	188 (64.83)	247 (61.8)	4.23	0.04^∗^
Q43: I obtain support from a group of caring people	82 (74.54)	207 (71.37)	289 (72.3)	0.39	0.52
Q44: I settle conflicts with others through discussion and compromise	78 (70.91)	207 (71.37)	285 (71.3)	0.01	0.92
*Mean (%)*	66.7	70.5	69.5		
Stress management					
Q45: I obtain sufficient sleep	49 (44.54)	148 (51.03)	197 (49.3)	1.34	0.24
Q46: I get enough time for leisure each day	48 (43.63)	147 (50.68)	195 (48.8)	1.58	0.20
Q47: I believe those effects in my life that I cannot modify	57 (51.81)	166 (57.24)	223 (55.8)	0.95	0.32
Q48: I focus on enjoyable feelings at bedtime	56 (50.90)	152 (52.41)	208 (52)	0.07	0.78
Q49: I use precise ways to manage my stress	55 (50)	150 (51.72)	205 (51.3)	0.09	0.75
Q50: I pose time between job and play	54 (49.09)	139 (47.93)	193 (48.3)	0.04	0.83
Q51: I perform leisure or contemplation for at least 15 to not more than 20 min a day	48 (43.63)	126 (43.44)	174 (43.5)	0.00	0.97
Q52: I pace myself to avoid exhaustion	57 (51.82)	141 (48.62)	198 (49.5)	0.32	0.56
*Mean (%)*	48.2	50.4	49.8		

Values for smoking and nonsmoking medical students are expressed as *n* (%). ^∗^*p* < 0.05.

**Table 2:** Comparison between health-promoting behaviors of smoking and nonsmoking medical students (*n* = 400) by the chi-square test.

Subscale	Smoking (*n* = 110) *N* (%)	Nonsmoking (*n* = 290) *N* (%)	Total *N* (%)	*X* ^2^	*p* value
Health responsibility					
Q1: I examine my body shape monthly for physical alterations/danger marks	19 (17.27)	72 (24.82)	91 (22.8)	2.59	0.11
Q2: I record any odd signs or marks to a physician or additional health professionals	51 (46.36)	153 (52.75)	204 (51)	1.30	0.25
Q3: I look for guidance or advice when necessary	79 (71.81)	189 (65.17)	268 (67)	1.59	0.20
Q4: I ask health professionals to realize their advice	78 (70.91)	171 (58.96)	249 (62.3)	4.84	0.03^∗^
Q5: I obtain a second judgment when asked about the opinion supplied by health professionals	74 (67.27)	185 (63.79)	259 (64.8)	0.42	0.51
Q6: I seek data from health professionals concerning how to obtain fine care of myself	55 (50)	120 (41.37)	175 (43.8)	2.40	0.12
Q7: I discuss health fears with health professionals	40 (36.36)	131 (45.17)	171 (42.8)	2.53	0.11
Q8: I follow TV programs about developing health	40 (36.36)	113 (38.96)	153 (38.3)	0.23	0.63
Q9: I attend learning programs on individual health care	39 (35.45)	109 (37.58)	148 (37)	0.15	0.69
*Mean (%)*	48	47.6	47.9		
Physical activity					
Q10: I pursue a designed exercise program	23 (20.91)	49 (16.89)	72 (18)	0.86	0.35
Q11: I train forcefully at least for 20 min for more than twice a week, for example, jogging, bicycling, climbing stairs, dancing, swimming	25 (22.72)	81 (27.93)	106 (26.5)	1.12	0.29
Q12: I practice light-to-moderate physical activity, for example, continue walking between 30 and 40 minutes at least five times a week	27 (24.54)	83 (28.62)	110 (27.5)	0.66	0.41
Q13: I engage in entertaining PA, such as bicycling and swimming	25 (22.72)	67 (23.10)	92 (23)	0.01	0.93
Q14: I perform stretching exercises more than three times a week	24 (21.82)	59 (20.34)	83 (21)	0.10	0.74
Q15: I do exercise through normal daily activities, for example, jogging and using stairs	46 (41.82)	122 (42.06)	168 (42)	0.00	0.96
Q16: I inspect my heart rate while exercising	23 (20.91)	54 (18.62)	77 (19.3)	0.26	0.60
Q17: I attain my target pulse rate while exercising	29 (26.36)	65 (22.41)	94 (23.5)	0.69	0.40
*Mean (%)*	24.8	25	25.1		
Nutritional habits					
Q18: I select a diet poor in fat whether saturated or unsaturated	33 (30)	86 (29.65)	119 (29.8)	0.00	0.94
Q19: I restrict the utilization of sugars and sweets	37 (33.64)	100 (34.48)	137 (34.3)	0.02	0.87
Q20: I consume at least six to less than 12 servings of bread, pasta, and rice daily	42 (38.18)	118 (40.69)	160 (40)	0.21	0.65
Q21: I consume at least two to less than five servings of fruit daily	41 (37.27)	105 (36.21)	146 (36.5)	0.04	0.84
Q22: I consume from three to not more than five servings of vegetables daily	38 (34.54)	117 (40.34)	155 (38.8)	1.13	0.29
Q23: I consume from two to not more than three servings of milk and cheese daily	38 (34.54)	118 (40.69)	156 (39)	1.26	0.26
Q24: I consume at least two to not more than three servings of eggs, meat, nuts, poultry, beans, and fish daily	49 (44.54)	136 (46.89)	175 (43.8)	0.17	0.67
Q25: I examine labels to discover nutrients, fats, and sodium in enclosed food	42 (38.18)	125 (43.10)	167 (41.8)	0.79	0.37
Q26: I have breakfast	63 (57.27)	171 (58.96)	234 (58.5)	0.09	0.76
*Mean (%)*	38.7	41.2	40.3		

Values for smoking and nonsmoking medical students are expressed as *n* (%). ^∗^*p* < 0.05.

**Table 3:** Comparison of subscale scores of the HPLBs between smoking and nonsmoking medical students (*n* = 400) using *t*-test.

Health promotion lifestyle profile	Smoking (*n* = 110)	Nonsmoking (*n* = 290)	Mean difference	*t*-test	*p* value
Health-promoting behaviors					
Health responsibility (nine questions)	22.29 ± 4.82	23.42 ± 14.45	-1.13	0.80	0.21
Physical activity (eight questions)	15.42 ± 5.02	15.40 ± 5.26	0.02	0.03	0.48
Nutritional habits (nine questions)	20.67 ± 4.88	21.45 ± 4.84	-0.78	1.43	0.07
Well-being habits					
Spiritual growth (nine questions)	27.16 ± 6.70	28.06 ± 5.75	-0.91	1.33	0.09
Interpersonal relations (nine questions)	25.58 ± 5.08	26.82 ± 5.66	-1.24	2.01	0.02^∗^
Stress management (eight questions)	20.04 ± 4.63	20.47 ± 5.07	-0.43	0.77	0.21
Total Health-Promoting Lifestyle Profile-II scale (52 questions)	131.17 ± 20.60	135.65 ± 24.52	-4.48	1.70	0.04^∗^

The mean values for smoking and nonsmoking medical students are given. ^∗^*p* < 0.05.

## Data Availability

All the utilized data to support the findings of the current study are included in the article.
